# A Case Report of Axial Torsion of Meckel’s Diverticulum: An Acute Abdomen Without Mechanical Bowel Obstruction in a Child

**DOI:** 10.7759/cureus.69235

**Published:** 2024-09-11

**Authors:** Yara Chaaban, Rawan Alhalabi, Muhammad Eyad Ba'Ath

**Affiliations:** 1 Pediatrics, American Hospital Dubai, Dubai, ARE; 2 Pediatric Surgery, University of Sharjah, Sharjah, ARE

**Keywords:** acute abdomen in children, axial torsion, bowel obstruction, complicated meckel's diverticulum, diverticular bowel obstruction, giant meckel’s diverticulum, meckel´s diverticulum, meckel's diverticulum complications, small-bowel obstruction, surgical acute abdomen

## Abstract

Meckel’s diverticulum is the most common congenital anomaly of the gastrointestinal tract. It is commonly silent but can cause multiple complications. The rarest presentation of Meckel’s diverticulum is axial torsion around its base without involving the main gut lumen. This can lead to acute abdomen without bowel obstruction. Here we present a case of an eight-year-old boy who was found to have a huge Meckel diverticulum with axial torsion causing necrosis of the cyst without involving the bowel's main lumen. This paper discusses potential diagnostic and therapeutic pitfalls of the axial torsion of an MD.

## Introduction

Meckel’s diverticulum (MD) is the most common congenital anomaly of the gastrointestinal tract. It results from the incomplete obliteration of the vitelline duct leading to the formation of a true diverticulum of the small intestine [1]. MD exists in 2% of the general population and is usually clinically silent [2]. While MD is commonly associated with complications such as gastrointestinal bleeding, inflammation, volvulus, and bowel obstruction or perforation, its rarest presentation is axial torsion around its base, which does not involve the main gut lumen. This unique form of torsion can lead to an acute abdomen without the typical signs of bowel obstruction, making it a diagnostic challenge. This case highlights the importance of considering axial torsion of MD in the differential diagnosis of acute abdomen, especially in pediatric patients, as prompt recognition and surgical intervention are key to avoiding complications. By sharing this case, we aim to raise awareness of this rare complication, contributing to improved diagnostic accuracy and clinical outcomes.

## Case presentation

**Figure 1:**
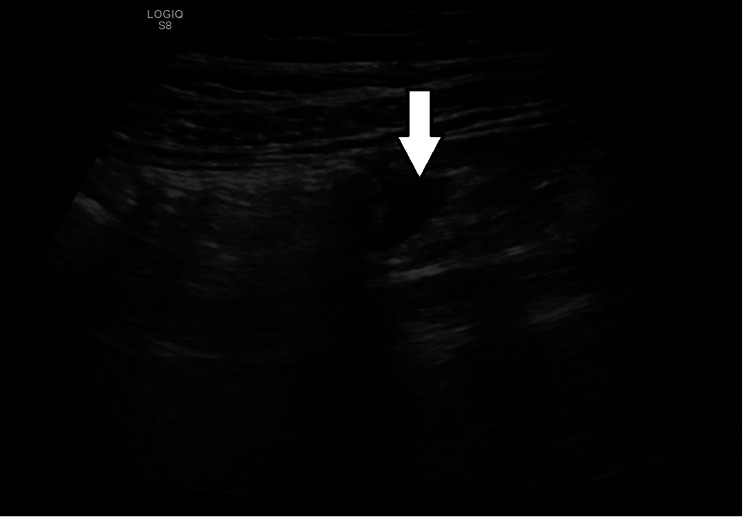
Abdominal Ultrasound Small pocket of free fluid noted in the right iliac fossa (white arrow).

**Figure 2:**
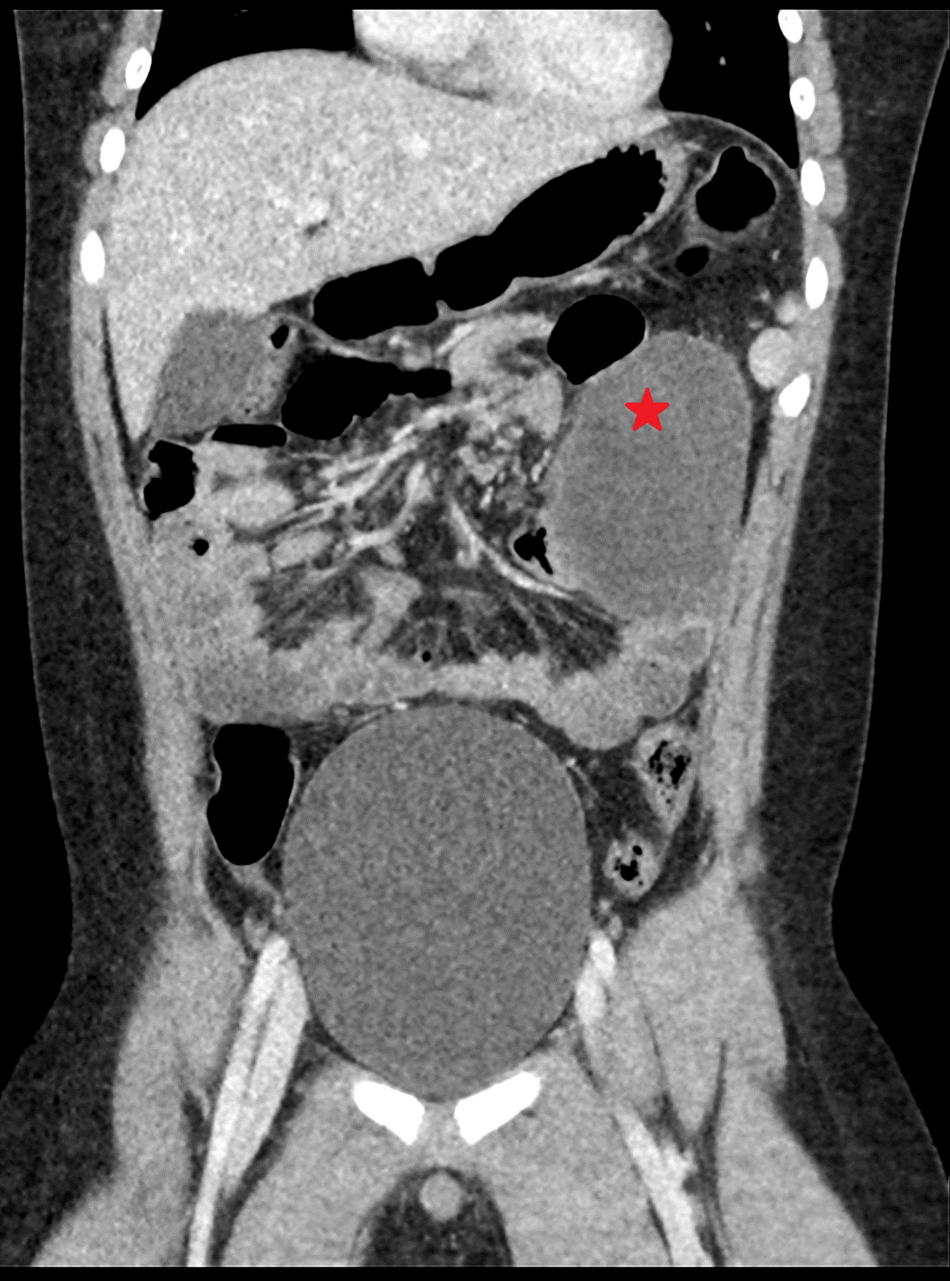
CT Scan - Abdomen Coronal CT showing upper abdominal small intestinal loop dilatation which is more prominent on the left side (red star), measuring about 48 mm in diameter and containing a single air-fluid level.

**Figure 3:**
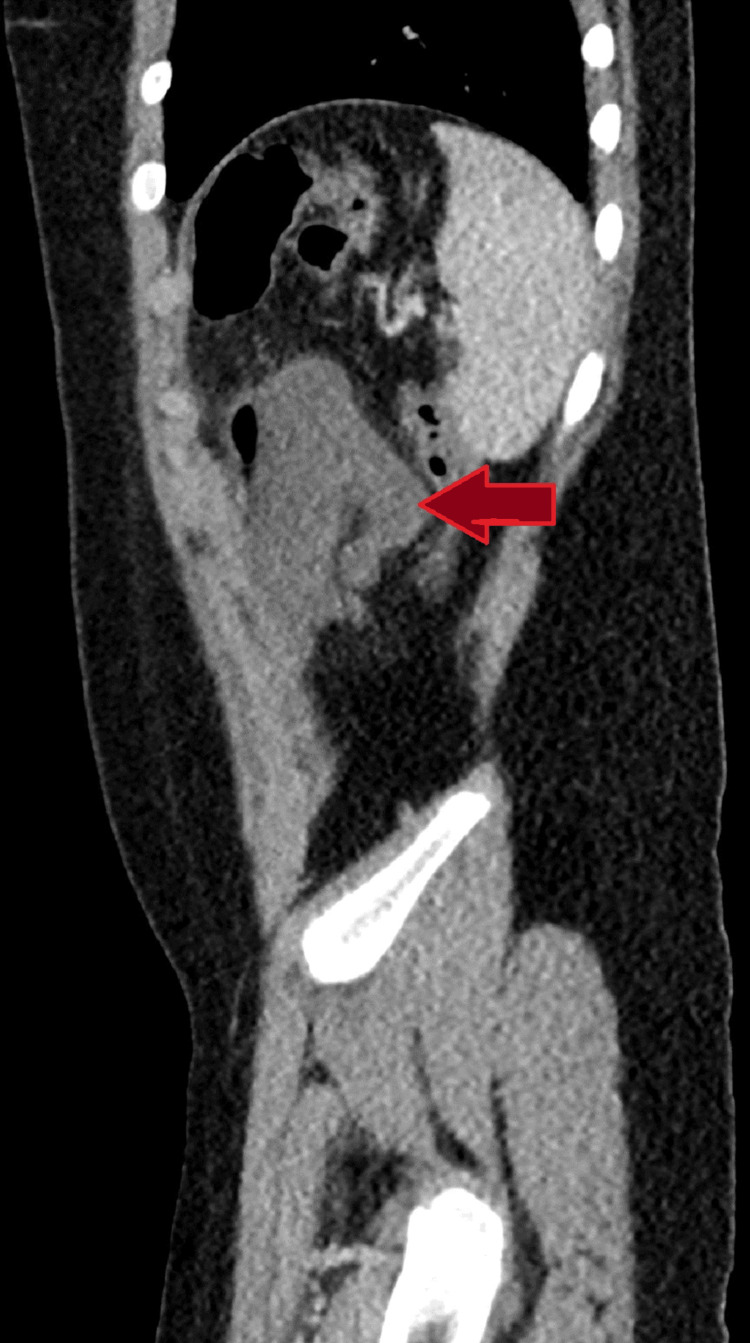
CT Scan - Abdomen Sagittal plane of CT demonstrates dilatation of a small intestinal loop in the central upper abdomen measuring about 28 mm in diameter (red arrow).

An eight-year-old male child presented to the emergency room with a 24-hour history of diffuse acute abdominal pain, fever, vomiting, and two loose stools. An abdominal ultrasound showed a small pocket of free fluid in the right iliac fossa (Figure 1), and a non-visualized appendix due to bowel gases. Computerized tomography (CT) revealed dilated central and left lateral abdominal small intestinal loops with left-sided single air-fluid level suggesting closed loop obstruction, and no evidence of intestinal ischemia or perforation (Figures 2, 3). The patient was stabilized with fluids and a laparotomy was performed.

**Figure 4:**
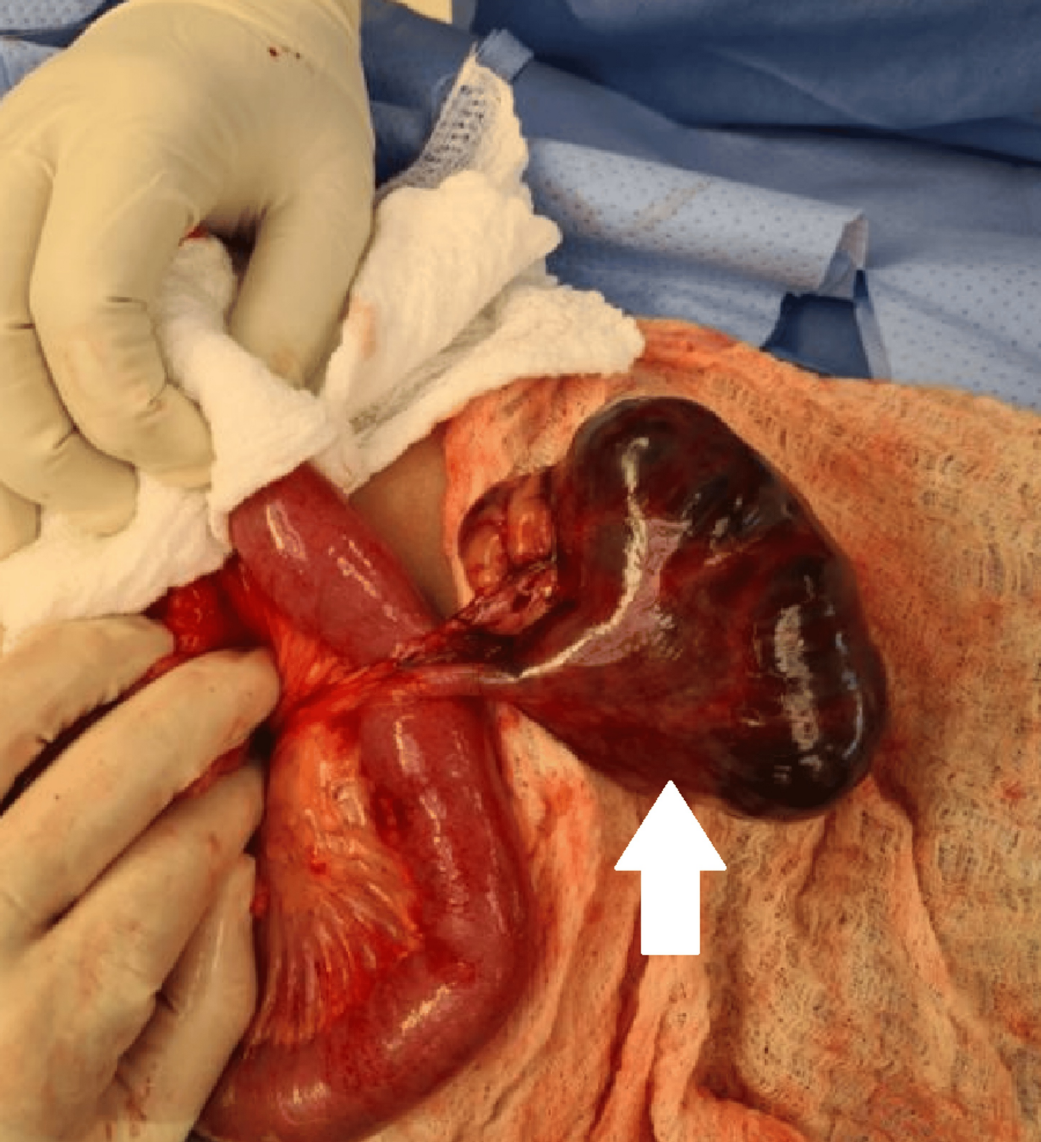
A Necrotic Meckel’s Diverticulum Cyst The necrotic Meckel’s diverticulum cyst (white arrow) follows a torsion around the base that does not involve the small bowel main lumen. The small bowels shown in the figure are normal.

Laparotomy revealed a huge Meckel diverticulum cyst almost 10 cm in diameter with a very narrow twisted pedicle. As shown in Figure 4, there was axial torsion around the pedicle of the MD causing necrosis of the pedicle and cyst without involving the bowel's main lumen.

The large air-fluid level loop on the CT scan turned out to be the cyst (Figures 2, 3). The diverticulum was necrotic; however, the bowel was viable and healthy and there was no bowel obstruction. The appendix and the rest of the bowel appeared to be normal. The cyst and pedicle were resected along with a 5 cm length of the ileum, adjacent to the cyst's base. Then end-to-end ileo-ileal anastomosis was performed. The histopathological study confirmed the diagnosis of a measured 9.5 x 8 cm Meckel diverticulum with hemorrhagic necrosis. The patient’s recovery was uncomplicated, and he was discharged on the fourth postoperative day with routine follow-up.

## Discussion

Axial torsion is the rarest complication of MD [2]. The rotation occurs along the diverticulum’s axis at its base without the involvement of the attached ileal loop or ileal mesentery. Subsequently, compromise of the vascular supply and eventually necrosis might occur without bowel obstruction.

The presenting symptom for axial torsion of the Meckel’s diverticulum is usually pain, mainly in the lower abdomen. When the tortured MD becomes necrotic and perforates, peritonitis and even sepsis might occur. The mobility of the diverticulum could contribute to misdiagnosis as the pain could be located anywhere in the abdomen. In our case, the maximum tenderness was in the left upper quadrant.

Abdominal ultrasound is often inconclusive, while CT may show an inflamed diverticulum or misinterpret the distended cyst as an obstructed bowel loop. If the diverticulum is small, no distended bowel loops may be visible on CT. MD may appear as a cystic, tube-like, non-peristaltic structure on ultrasonography [3]. In our case, the CT scan mistakenly identified the distended MD cyst as a closed-loop obstruction, which was inconsistent with the patient's history of diarrhea.

The mechanism underlying the isolated torsion of Meckel’s diverticulum is still obscure although many factors may be associated with the axial torsion of Meckel’s diverticulum. The anatomical specifications of MD such as length and breadth and the base diameter of the diverticulum are important predisposing factors. The risk of torsion increases with longer and larger Meckel’s diverticulum with a narrow base [1,3,4]. Attachment of the distal end of Meckel’s diverticulum to the umbilicus due to retained fibrous vitelline band increases the chances of torsion [5]. Another potential risk factor is primary neoplasm arising within Meckel’s diverticulum, which is very rare [6].

## Conclusions

Axial torsion of MD is an extremely rare complication. An enlarged MD along with its narrow base could be a risk factor. It is difficult to diagnose axial torsion of MD with imaging alone, especially in the absence of bowel obstruction. Hence, it is important to maintain a high index of suspicion and to confirm the diagnosis by doing an exploratory laparotomy. Treatment of choice for such cases is surgical excision of MD and primary anastomosis.
